# Advances and trends in microbial production of polyhydroxyalkanoates and their building blocks

**DOI:** 10.3389/fbioe.2022.966598

**Published:** 2022-07-19

**Authors:** Qiang Gao, Hao Yang, Chi Wang, Xin-Ying Xie, Kai-Xuan Liu, Ying Lin, Shuang-Yan Han, Mingjun Zhu, Markus Neureiter, Yina Lin, Jian-Wen Ye

**Affiliations:** ^1^ Key Laboratory of Plateau Ecology and Agriculture, Qinghai University, Xining, QH, China; ^2^ School of Biology and Biological Engineering, South China University of Technology, Guangzhou, China; ^3^ Institute for Environmental Biotechnology, Department of Agrobiotechnology, University of Natural Resources and Life Sciences, Tulln, Austria

**Keywords:** polyhydroxyalkanoates, synthetic pathway, metabolic engineering, PHA synthase, microbial production

## Abstract

With the rapid development of synthetic biology, a variety of biopolymers can be obtained by recombinant microorganisms. Polyhydroxyalkanoates (PHA) is one of the most popular one with promising material properties, such as biodegradability and biocompatibility against the petrol-based plastics. This study reviews the recent studies focusing on the microbial synthesis of PHA, including chassis engineering, pathways engineering for various substrates utilization and PHA monomer synthesis, and PHA synthase modification. In particular, advances in metabolic engineering of dominant workhorses, for example *Halomonas, Ralstonia eutropha, Escherichia coli* and *Pseudomonas,* with outstanding PHA accumulation capability, were summarized and discussed, providing a full landscape of diverse PHA biosynthesis. Meanwhile, we also introduced the recent efforts focusing on structural analysis and mutagenesis of PHA synthase, which significantly determines the polymerization activity of varied monomer structures and PHA molecular weight. Besides, perspectives and solutions were thus proposed for achieving scale-up PHA of low cost with customized material property in the coming future.

## Introduction

Polyhydroxyalkanoates (PHAs) is a series of polyesters synthesized by different microbes (Steinbüchel, 2001), which have been widely used as bio-plastics for replacing petrol-based plastic due to their outstanding biodegradability and biocompatibility. Accordingly, PHA can be divided into three categories (Sudesh et al., 2000) including short-, medium- and long- chain-length PHAs, namely SCL-, MCL- and LCL-PHA, respectively. Of which, the monomers of SCL-, MCL- and LCL-PHA generally contain 2–5, 6–14 and over 15 carbon atoms, respectively. Because of the competitive material properties, PHA has attracted growing attentions of commercial interests in different application areas, such as medical implant (Chen and Wu, 2005), cosmetic beads (Choi et al., 2020), packaging (Chen and Patel, 2012), agricultural film (Chen, 2009), textile (Chen, 2009), feeding additives (Chen, 2009) and so on. In the past decades, intensive efforts have been made to generate various PHA productions consisting of diverse polymerized units with different carbon-chain-length and structures by genetically modified bacterial (Chen and Jiang, 2017), such as *Halomonas spp.* (Tan et al., 2011; Fu X. Z. et al., 2014)*, Ralstonia eutropha* (Antonio et al., 2000; Raberg et al., 2018; Xiong et al., 2018)*, Escherichia coli* (Park et al., 2001; Linares-Pastén et al., 2015; Sudo et al., 2020)*, Pseudomonas* spp (Chanasit et al., 2016; Liang et al., 2020; Li M. et al., 2021) and so on (Hyakutake et al., 2014; Tariq et al., 2015). Therefore, over 150 types of PHAs have been obtained including homopolymers (PHB, poly-3-hydroxybutyrate) (Tan et al., 2011), random- and/or block- copolymers such as poly(3-hydroxybutyrate-*co*-4-hydroxybutyrate) (P34HB), poly(3-hydroxybutyrate-*co*-3-hydroxyvalerate) (PHBV) (Fu X. Z. et al., 2014), poly(3-hydroxybutyrate-*co*-3-hydroxyhexonate) (PHBHHx) (Park et al., 2001), etc. (Li M. et al., 2021). To date, many building blocks, including rational designed enzymes (Chek et al., 2019; Lim et al., 2021), fine-tuned metabolic pathways towards monomer synthesis (Pacholak et al., 2021) and genetically engineered chassis of predominant PHA accumulation performance (Liang et al., 2020; Ye and Chen, 2021), have been developed for sufficient PHA synthesis using a variate of substrates.

In particular, scale-up industrial production lines for various PHA manufacturing have been recently launched or established by several companies, for example, MedPHA (operating production line of 1,000 ton/year PHB and/or P34HB, China) (Obruča et al., 2022), PhaBuilder (10,000 ton/year, under construction, China) (Yang et al., 2010), Tianan (3,000 ton/year PHBV, China) (Modi et al., 2011), Tepha (P4HB for medical uses, United States) (Martin and Williams, 2003), Danimer Scientific (6,000 ton/year PHBHHx, United State) (Mehrpouya et al., 2021), Keneka (5,000 ton/year PHBHHx, Japan) (Tanaka et al., 2021). However, the production cost of PHA still challenges for wide range commercial uses. Therefore, many solutions have been proposed and developed to reduce the industrial cost of PHA, including high cell density fermentation based on optimized feeding solution (Silva et al., 2017), non-sterile open fermentation process based on recombinant halophiles (Tan et al., 2011), cell factory engineering for effective utilization of low-cost carbon sources (Murugan et al., 2017; Panich et al., 2021), carbon fixation engineering for the improved conversion rate from glucose to PHA (Salehizadeh et al., 2020), co-production of PHA and value-added chemicals (Lan et al., 2016; Li et al., 2016) and so on.

Therefore, this study summarized recent advances of various PHA production and industrial trends thereof. Additionally, major building blocks, including representative workhorses, metabolic pathways and critical enzymes, for PHA synthesis have been reviewed and discussed. This study provides an entire landscape of PHA productions powered by synthetic biology, as well as perspectives focusing on cost-effective PHA manufacturing in the coming future.

## Workhorses for PHA production

### *Halomonas bluephagenesis* TD01

*Halomonas bluephagenesis* TD01 (*H. bluephagenesis*), a natural PHB producer isolated from salt lake (Tan et al., 2011), has been recently developed as a versatile chassis for PHA productions and value added chemicals, which exemplifies a cost-effective biomanufacturing paradigm based on next generation industrial biotechnology (NGIB) enabling non-sterile open fermentation process under high salt and high pH condition (Ye and Chen, 2021). Currently, the genetically reprogrammed *H. bluephagenesis* can produce various PHA polymers, including PHB (Tan et al., 2011), PHBV (Fu X. Z. et al., 2014), P34HB (Chen et al., 2017) and PHBP (poly-3-hydroxybutyrate-*co*-3-hydroxypropionate) (Jiang et al., 2021) using glucose, starch, gluconate and structural related carbon sources for corresponding monomer synthesis whenever necessary, for example, 4HB from γ-butyrolactone (GBL), 3HP from 1,3-propanediol, 3HV from propionate, etc. Notably, pilot-scale production of PHB and P34HB have succeeded in a 5,000-L bioreactor, yielding up to 100 g/L dry cell mass (DCM) containing 60–70 wt% PHA content with over 30% cost reduction (Ye et al., 2018). Besides, engineering electron transport system could significantly improve the supplementation of NADH (Ling et al., 2018), overexpression of Vitreoscilla hemoglobin (VHb) protein led to improved oxygen uptake efficiency (Ouyang et al., 2018), deficiency of outer membrane synthesis enabled sufficient production yield of PHA from glucose and simplified cell lysis (Wang Z. et al., 2021), manipulation of cell morphology also resulted in self-flocculation separation process (Ling et al., 2019). Moreover, different genetic parts and tools have been established allowing for rational engineering of *H. bluephagenesis* (Zhang et al., 2020). These efforts have proved successful in building a high-performing workhorse for PHA production based on NGIB. Additionally, many other *Halomonas* strains were also successfully developed for PHA synthesis, such as *Halomonas campanesis* LS21 (Yue et al., 2014), *Halomonas elongate* DSM2581 (Ilham et al., 2014), *Halomonas pacifica* ASL10 (Abd El-malek et al., 2020) and so on, illustrating the great potential of halophiles used as PHA producers.

### Ralstonia eutropha

*Ralstonia eutropha* H16 (*Cupriavidus necator*) is a well-studied PHA producer from glucose, glycerol, palm oil and other fatty acids (FAs) (Murugan et al., 2017). In addition to short chain length PHA synthesis, *R. eutropha* H16 has been engineered to produce varied copolymers consisting of SCL-monomer (3HB) and MCL-monomers, such as 3HHx, 3HO (3-hydroxyoctanoate), 3HDD (3-hydroxydodecanoate) and so on (Antonio et al., 2000). In previous studies, genetic editing tools for chromosomal engineering was established based on CRISPR/Cas9 system and Cre/LoxP integrase system (Park et al., 2001). An electroporation approach was developed in recombinant *R. eutropha* H16 allowing for sufficient and high-through clone construction (Xiong et al., 2018). More importantly, over 200 g/L DCM with over 70 wt% PHA accumulation can be obtained by *R. eutropha* H16 and its derivates during fed-batch fermentation conducted in the lab- (<10-L) and/or pilot- (>100-L) scale bioreactors under strictly sterilized conditions (Ryu et al., 1997). Moreover, industrial productions of PHB, PHBV and PHBHHx based on recombinant *R. eutropha* H6 have been achieved by several companies. Therefore, *R. eutropha* H16 is expected to be a prominent chassis for PHA productions, especially for PHBHHx, however, high production cost remains challenging (Raberg et al., 2018).

### Escherichia coli

*Escherichia coli* (*E. coli*), such BL21, JM109, etc., are well-studied model chassis that have clear genetic background and effective genetic tools for cell factory engineering of varied purposes, such as PHA biosynthesis. Even though *E. coli* is not a natural PHA producer, the heterogonous expression of *phaCAB* gene cluster from *R. eutropha* could efficiently boost carbon flux from pyruvate towards PHB synthesis. Therefore, intensive studies focusing on CO_2_ fixation (Lee et al., 2021), pathway engineering (Chen and Jiang, 2017) and feeding solution design of fed-batch fermentation (Yang et al., 2014) have been performed to generate enhanced production yield of PHB. Besides, *E. coli* is an ideal workhorse for studying the novel-type PHA synthesis, such as copolymers of 3HB and lactate, glycolic acid, 4-hydroxybutyrate, 5-hydroxyvalerate and other monomers with functional groups (Scheel et al., 2021). Specifically, the DCM and PHA content reached up to 194 g/L and 73 wt% by recombinant *E. coli* grown in fed-batch fermentation condition (JONG-IL CHOI, 1998), which shows promising performance in PHA accumulation.

### Pseudomonas

*Pseudomonas*, including *P. putida* KT2440, *P. entomophila*, etc. have been recently engineered to be dominant producers of PHA copolymers consist of 3HB and MCL- and LCL-3HAs due to their strong FAs metabolism involved in β-oxidation cycle and *de novo* FAs synthesis pathways. Currently, PHA copolymers are composed of 3HB, 4HB, 3HV, 3HHx, 3HHp (3-hydroxyheptanoate), 3HO (3-hydroxyoctanoate), 3HD (3-hydroxydecanoate), etc. could be obtained by metabolically engineered *Pseudomonas* strains (Prieto et al., 2016). Many PHA synthases able to polymerize MCL- and LCL-3HA into polymers were thus identified from different *Pseudomonas* strains (Chung et al., 2011; Li et al., 2019; Tan et al., 2020; Li M. et al., 2021). Notably, an effective platform was developed for producing full spectrum of PHAs, which contain SCl-, MCL, LCL- 3HAs and monomers carrying carbon-carbon double bones, with over 90% increase in production yield based on recombinant *P. entomophila* (Li M. et al., 2021). Moreover, higher DCM, reaching over 70 g/L, was also achieved by *Pseudomonas* leveraging fed-batch fermentation process optimization (Cerrone et al., 2014). These efforts demonstrate proven success in scalable tailor-made PHA synthesis of varied functions by reprogrammed *Pseudomonas*.

Additionally, various attempts have been carried out to achieve PHA synthesis based on different hosts, such as *Alcaligenes* (H W Ryu 1996), *Bacillus* (Sathiyanarayanan et al., 2013), *Burkholderia* (Miranda De Sousa Dias et al., 2017), *microalgae* (Costa et al., 2019), *Salinivibrio* (Van Thuoc et al., 2019; Van Thuoc et al., 2020), *Marinobacterium* (Wang et al., 2022), *Vibrio alginolyticus* (Li H. F. et al., 2021) and so on, using diverse carbon sources including sucrose, propionate, carbon dioxide, volatile fatty acids, etc. It is important to note that the highest resultant DCM reached up to 281 g/L with 232 g/L PHB accumulation by *Alcaligenes eutrophus*, a natural PHB producer of high cell density growth and effective PHA accumulation, during a 74 h fed-batch fermentation conducted in a 60-L bioreactor (H W Ryu 1996).

## Metabolic pathways for PHA synthesis

**FIGURE 1:**
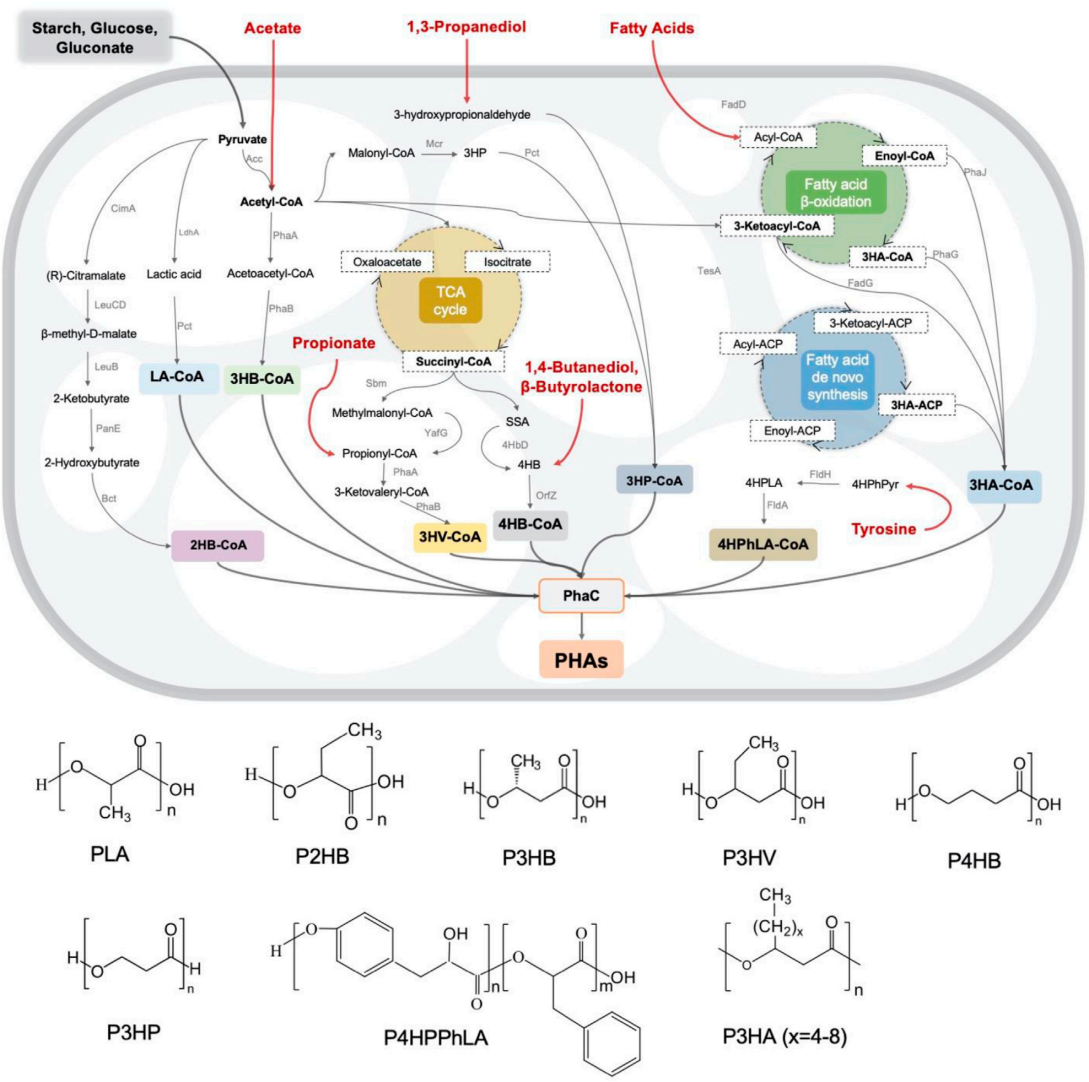
Metabolic pathways and monomer structures of different microbial synthesized PHAs.

The biosynthesis pathways of most PHA monomers from varied carbon sources like glucose, fatty acids, etc. are mainly related to essential carbon metabolic pathways, such as glycolysis, β-oxidation and *de novo* fatty acid synthesis (Figure 1). Besides, using structurally related carbon sources as precursors is an alternative strategy to generate diverse PHA copolymers consist of different monomers, including 4HB from γ-butyrolactone (GBL)/1,4-butanediol (BDO), 3HV from propionate, 3HP from 1,3-propionediol (PDO), middle- and long-chain length 3HA from different fatty acids with corresponding carbon atoms and so on, which have significant impact on the material property (Chen et al., 2016). Therefore, a wide variety of PHA homo- and co-polymers can be obtained by engineered microbes by feeding customized feedstocks (Figure 1).

### PHA from structure-unrelated carbon sources

Glucose is a widely used feedstock in biomanufacturing. Similarly, intensive studies have been carried out for generating different PHA using glucose as the sole carbon source by metabolically engineered microorganisms. To date, many metabolic pathways have been mined and refined as significant building blocks for rewiring glucose-derived fluxes towards various monomers, such as converting pyruvate into2HB-CoA (Park et al., 2012c) and LA-CoA (Park et al., 2012b), acetyl-CoA into 3HB-CoA and 3HP-CoA (Meng et al., 2015), succinyl-CoA into 3HV-CoA (Bhatia et al., 2015) and 4HB-CoA (Lv et al., 2015), respectively. Interestingly, starch (Yang et al., 2020), volatile fatty acids like acetate (Yang et al., 2019), waste gluconate (Ciesielski et al., 2010), the byproduct of glucose processing, were also used to culture engineered *Halomonas* and *Pseudomonas* to achieve cost-effective PHA productions (Figure 1).

In addition to glucose, building blocks for many other carbon sources metabolism, such as glycerol, sucrose, xylose, C1 compounds, etc., have been constructed to synthesize PHA. Specifically, the highest PHA accumulation, reaching 38.9 wt% with 0.34 g/L/h of productivity have been achieved by engineered *P. putida* KT2440 (Borrero-de Acuna et al., 2021). Fu et al. produced MCL-PHA also could be obtained by grown on chemical-grade glycerol (PG) and biodiesel-derived waste glycerol (WG) as sole carbon sources (Fu J. et al., 2014). Moreover, recombinant strains including *P. putida S12* and *R. eutropha* harboring expression vessel containing isomerase (XylA) and xylulokinase (XylB) have been constructed by Meijnen et al. (2008) and Kim et al. (2017), respectively, to produce PHA using xylose as sole carbon source (Meijnen et al., 2008). Similarly, a sucrose-favored *P. putida* strain was also developed for PHA synthesis from sucrose only (Hobmeier et al., 2020). More importantly, due to the growing interests of global carbon neutral, many bacterial like *P. furiosus* and *R. eutropha* B8562 were engineered to produce PHA polymers containing 3HP and 3HB units, respectively, using CO_2_ as carbon source (Volova et al., 2006; Keller et al., 2013). Besides, biosynthesis pathways for PHA synthesis from CH_4_ were also established based on many hydrogen-oxidizing bacteria (Khosravi-Darani et al., 2013). In summary, metabolic engineering of microbes is able to achieve targeted PHA synthesis from different structure-unrelated carbon sources.

### PHA from structure-related carbon sources

For most MCL- and LCL-PHA synthesis, supplementation of structure-related fatty acids in the medium is a commonly used strategy to grow recombinant cells with defected β-oxidation cycle or reprogramed *de novo* fatty acids synthesis pathways (Gutierrez-Gomez et al., 2019). For instance, a wide range of PHA copolymers composed of 3HB and MCL-/LCL-3HA units containing carbon atoms numbered from 6 to 18, even with carbon-carbon double bone, have been achieved by engineered *Pseudomonas*, yielding over 100% increase of production titer (Yao J, 1999). Besides, many short chain length (SCL) PHA units were also produced from structure-related carbon sources used as precursors, such as 4HB synthesis from 1,4-propanediol (PDO) and β-butyrolactone (GBL) (Cavalheiro et al., 2012), 3HP synthesis from 1,3-propionediol (PDO) (Zhou et al., 2011), 5HV synthesis from 1,5-pentanediol (Yan et al., 2022), as well as functional group monomer like 4HPhLA synthesized from tyrosine (Yang et al., 2018), etc. Recently, high production yield of P34HB with 4HB molar ratio from 5 mol% to 26 mol% has been achieved by recombinant *H. bluephagenesis* based on NGIB platform, which also demonstrated the success in scale-up production of low cost conducted in 5-to-200 m³ fermenters (Ling et al., 2018). Notably, Lee et al. (2021) used engineered *Escherichia coli* to synthesize aromatic polyester, P(3HB-*co*-D-phenylacetate), from tyrosine, of which the molar ratio of D-phenylacetate monomer reaches up to 47.7 mol% (Yang et al., 2018). Moreover, tailor-made copolymers, as well as block copolymers, consisting of two, three and even more units could be easily obtained by designing the supplementation formula of target precursors and feeding strategy thereof (Yu et al., 2020).

## Engineering tools and strategies for sufficient PHA synthesis

In addition to the biosynthesis pathway construction for diverse PHA productions, many metabolic engineering tools including high resolution gene expression tuning (Ye et al., 2020), high throughput library construction (Zhou et al., 2015; Young et al., 2018), constitutive and inducible promoter design (Shen et al., 2018; Ma et al., 2020) and so on have been developed for constructing effective PHA producing strains. Moreover, a carbon fixation of CO_2_ was established in *E. coli* to generate an increased bioconversion rate of glucose towards PHB (Lin et al., 2015). Modulating the NADH levels and its regeneration pathways could also show proven effects on PHA accumulation in both *E. coli* and *Halomonas* strains with PHA content increased up to 90 wt% (Ling et al., 2018). Interestingly, cell morphology control is an efficient strategy to obtain enhanced PHA accumulation with significantly improved substrate conversion rate (Wang X. et al., 2021). Manipulation of PHA granule size also demonstrated strong significance for downstream processing, which dramatically reduce the energy consumption of cell separation and PHA purification (Kourmentza et al., 2017). Therefore, the downstream-inspired engineering of microbes also displays great significance in cost-reduction for industrial PHA biomanufacturing.

## PHA synthase

PHA synthase (PhaC) is an important building block for PHA synthesis. Generally, there are four major types of PhaC, namely Class-I/II/III/IV (Możejko-Ciesielska and Kiewisz, 2016), which have been identified from different PHA producing strains (Table 1). Of which, Class-I, -III and IV generally show higher activity on short-chain-length (SCL) monomers (C3-C5) polymerization, while Class-II has higher specificity to medium- and long- chain-length (M/LCL) monomers containing 6–18 carbon atoms, namely C6-C18 (Chek et al., 2017). Specifically, most Class-I PHA synthases, such as PhaCs from *R. eutropha* (Ushimaru et al., 2014), *Alcaligenes latus* (Park et al., 2012a), *Aeromonas Caviae* and *Chromobacterium sp*. (Choi et al., 2020), not only show effective activity on SCL PHA accumulation including 3HB, 3HP, 4HB and 3HV units, but also display polymerization capability of MCL PHA like 3HHx (Antonio et al., 2000).

**TABLE 1:** Different PHA synthases identified from natural PHA producing strains.

Class	Source	Expression host	PHA	PHA content	DCM (g/L)	References
I	*Ralstonia eutropha*	*A. eutrophus* (NCIMB 11599)	PHB	83 wt%	281	Ryu et al. (1997)
I	*Ralstonia eutropha*	*C. necator* Re2133	P (3HB-*co*-18.5 mol% 3HHx)	52 wt%	1.1	Bhatia et al. (2019)
I	*Ralstonia eutropha*	*Ralstonia eutropha* PHB-4	P (3HB-*co*-5 mol% 3HP-*co*-10 mol% 5HV)	12 wt%	0.3	Chuah et al. (2013)
I	*Ralstonia eutropha*	*E. coli* JM109SGIK	P (3HB-*co*-7.89 mol% 4HB)	78 wt%	11.6	Wang et al. (2014)
I	*Ralstonia eutropha H16*	*Pseudomonas putida* KTOY08∆GC	P (3HB-*b*-80.31 mol% 4HB)	50 wt%	5.5	Hu et al. (2011)
I	*Ralstonia eutropha*	*E. coli*	P (3HB-*co*-84 mol% 3HP)	42 wt%	5	Meng et al. (2015)
I	*Aeromonas caviae*	*Ralstonia eutropha* PHB-4	P (3HB-*co*-35 mol% 3HV-*co*-3HHx)	80 wt%	7.1	Wang et al. (2014)
I	*Chromobacterium sp*	*E. coli*	P3HP	40 wt%	-	Linares-Pastén et al. (2015)
I	*Aeromonas caviae*	*Burkholderia* sp. USM (JCM15050)	P (3HB-*co*-34 mol% 3HV-*co*-6 mol% 3HHx)	86 wt%	1.5	Chee et al. (2012)
II	*Pseudomonas sp. 61–3*	*Pseudomonas entomophila*	P (3HB-*co*-14 mol% 3HPD)	60 wt%	9	Li et al. (2021b)
II	*Pseudomonas sp. 61–3*	*E. coli* W3110	P (11 mol% 3HHx-*co*-39 mol% 3HO-*co*-50 mol% 3HD)	4.8 wt%	1.7	Park et al. (2002)
II	*Pseudomonas sp. MBEL 6–19*	*E. coli* XL1-Blue	P (38.1 mol% PhLA-*co*-3HB)	55 wt%	13.9	Yang et al. (2018)
II	*Pseudomonas sp. MBEL 6–19*	*E. coli* XL1-Blue	P (88.2 mol%LA-*co-*11.8 mol%GA)	12.6 wt%	-	Choi et al. (2016)
II	*Pseudomonas*	*E. coli*	P (8.2 mol%GA-*co-*16.3 mol%GA-*co*-66.1 mol%3HB-*co*-9.4 mol%4HB)	72.89 wt%	19.6	Li et al. (2017)
II	*Pseudomonas mendocina*	*Pseudomonas mendocina*	P (3HB-*co*-3HO-*co*-3HD)	77 wt%	3.7	Chanasit et al. (2016)
II	*Pseudomonas oleovorans ATCC 29347*	*Pseudomonas oleovorans* ATCC 29347	mcl-PHA	63 wt%	18	Jung et al. (2001)
III	*Thiocapsa pfennigii*	*Pseudomonas putida* GPp104	P (3HB-*co*-3HV-*co*-15.4 mol% 4HV)	52 wt%	20	Gorenflo et al. (2001)
IV	*Bacillus cereus FA11*	*Bacillus cereus* FA11	P (3HB-*co*-6.49 mol% 3HV)	49 wt%	6.2	Tariq et al. (2015)
IV	*Bacillus cereus YB-4*	*E. coli* JM109	PHB	36 wt%	3.0	Hyakutake et al. (2014)
-	*Halomonas bluephagenesis TD01*	*Halomonas bluephagenesis* TD01	P (3HB-*co*-16.1 mol% 4HB)	61 wt%	82.6	Chen et al. (2017)
-	*Burkholderia sacchari DSM 17165*	*Burkholderia sacchari* DSM 17165	P (3HB-*co*-1.6 mol% 4HB)	73 wt%	72.9	Miranda De Sousa Dias et al. (2017)
-	*Cupriavidus malaysiensis USMAA2-4*	*Cupriavidus malaysiensis* USMAA1020	P (3HB-*co*-99 mol% 4HB)	92 wt%	50.4	Norhafini et al. (2019)

-Not classified PHA synthase.

Recently, many efforts have been made to modified the polymerization activity of PHA synthase, including protein structure analysis (Chek et al., 2017), mutagenesis (Zou et al., 2017) and fusion of functional domains from different PhaCs (Matsumoto et al., 2009), to generate high-performing PHA synthase. For instance, Kim et al. (2017) report the first crystal structure of *Ralstonia eutropha* PHA synthase at 1.8 Å resolution and structure-based mechanisms for PHA polymerization, RePhaC1 contains two distinct domains, the N-terminal (RePhaC1ND) and C-terminal domains (RePhaC1CD), and exists as a dimer (Kim et al., 2017). Furthermore, site-directed mutation was employed to generate PhaC mutants, namely PhaC_61-3_ and PhaC_1437_, based on PHA synthases from *Pseudomonas* sp. 61–3 and *Pseudomonas* sp. MBEL 6–19, respectively, which show wide substrate specificity to both SCL and M/LCL monomers (Yang et al., 2010), as well as monomers with a particular structure like benzene ring (Mizuno et al., 2018). Moreover, an artificial PHA synthase, PhaC_AR_, was constructed by hybridizing the C-terminal of PhaC_AC_ from *Aermonas Caviae* and N-terminal of PhaC_RE_ from *R. eutropha* (*Cupriavidus necator*), enabling effective accumulation for block copolymers containing 2-hydroxybutyrate (2HB) (Sudo et al., 2020);

## Conclusion and perspective

In this study, we highlighted the global trends of industrial PHA productions reported by different companies and start-up teams, and briefly summarized and discussed the advances of different building blocks focusing on PHA synthase, biosynthesis pathways of SCL-, MCL- and LCL-PHA, dominant PHA workhorses of industrial potential and optimization strategies for effective PHA synthesis. This study provides an overview of PHA biosynthesis from enzyme engineering, cell factory design, towards scale-up bio-manufacturing. However, more attempts are still required to achieve further cost-reduction and improved material properties of tailor-made PHAs against the petrol-based plastics.
